# Single-cell transcriptomics in metastatic breast cancer: mapping tumor evolution and therapeutic resistance

**DOI:** 10.3389/fgene.2025.1669741

**Published:** 2025-10-23

**Authors:** Xu Han, Xin Li, Ling Bai, Gangling Zhang

**Affiliations:** ^1^ Breast Center, Baotou City Cancer Hospital, Baotou, Inner Mongolia, China; ^2^ Operating Room, Baotou City Cancer Hospital, Baotou, Inner Mongolia, China

**Keywords:** ScRNA-seq, metastatic breast cancer, tumor heterogeneity, therapeutic resistance, precision oncology

## Abstract

Metastatic breast cancer (MBC) remains the primary cause of mortality in breast cancer patients, driven by tumor heterogeneity, cellular evolution, and therapy-resistant clones. Traditional bulk transcriptomics, although informative, fail to capture rare subpopulations and context-specific gene expression, which are crucial for understanding disease progression. Single-cell transcriptomics (SCT) has emerged as a transformative approach, enabling high-resolution analysis of individual cells to reveal tumor composition, lineage dynamics, and transcriptional plasticity. This review highlights how SCT reshapes our understanding of MBC by mapping tumor evolution, identifying cancer stem-like cells, and characterizing states of epithelial-mesenchymal transition. We explore how SCT reveals clonal and spatial heterogeneity, and how tumor microenvironment components, including immune, stromal, and endothelial cells, interact with cancer cells to support immune evasion and the formation of a metastatic niche. SCT also uncovers mechanisms of therapeutic resistance, including transcriptional reprogramming and the survival of drug-tolerant subpopulations. Integrating SCT with spatial transcriptomics and multi-omics platforms offers a comprehensive view of the MBC ecosystem and may uncover novel therapeutic targets. We further discuss the translational potential of SCT for biomarker discovery, liquid biopsy development, and precision oncology. We address current technical challenges and future directions for clinical application. SCT is poised to transform MBC research and guide next-generation therapeutic strategies.

## 1 Introduction

Metastatic breast cancer (MBC) continues to be a predominant cause of cancer-related death in women worldwide. In 2020, there were around 2.3 million new breast cancer cases and over 685,000 fatalities recorded globally (Zhang Y. et al., 2025). While merely 6%–10% of breast cancer patients exhibit *de novo* metastasis upon diagnosis, as many as 30% of early-stage cases ultimately advance to metastatic disease, frequently years following first therapy. The survival statistics for MBC are bleak, with a median overall survival of about 2–3 years; however, results differ based on molecular subtype and therapeutic accessibility (Anwar et al., 2025). MBC is characterized by the dissemination of malignant cells from the primary breast tissue to distant organs, such as the bones, lungs, liver, or brain. Apart from the considerable progress in the early detection and management of localized breast cancer, metastatic breast cancer remains predominantly incurable and imposes a disproportionate illness burden (Wang et al., 2025). The clinical management of metastatic breast cancer is further confounded by its intrinsic heterogeneity; patients may exhibit significantly varied molecular subtypes, treatment responses, and survival rates, even within the same histological classification. These limitations underscore the pressing need for more sophisticated molecular instruments to analyze the complexities of metastatic disease and inform targeted treatment (Zhang and Peng, 2025).

Historically, transcriptomic profiling of tumors has predominantly utilized bulk RNA sequencing (RNA-seq), which yields an averaged gene expression profile for a population of cells. This technique has yielded significant insights into tumor biology and categorization; however, it inadequately captures the cellular diversity within the tumor microenvironment (TME) (Ortega-Batista et al., 2025). Bulk RNA-sequencing often overlooks the roles of infrequent or functionally significant cell types, such as cancer stem cells, immunological infiltrates, and stromal components, which are frequently pivotal in metastasis and therapy resistance (Safarzadeh et al., 2025). Furthermore, geographical and temporal variability within the tumor and its metastatic microenvironments remains predominantly unexamined using standard transcriptome methodologies (Boggs, 2024).

Given these constraints, single-cell RNA sequencing (scRNA-seq) has emerged as a revolutionary method that may elucidate tumor complexity with unparalleled resolution (Sabit et al., 2025a). scRNA-seq facilitates the examination of gene expression at the single-cell level, enabling researchers to delineate the heterogeneous cellular ecology of MBC, identify unusual subpopulations, and track lineage trajectories during tumor progression (Kotsifaki et al., 2024). Recent scRNA-seq research has uncovered new insights into epithelial-mesenchymal transition (EMT), immune evasion, and drug resistance mechanisms in breast cancer metastasis. This method can potentially identify biomarkers that predict therapeutic response and inform the rational design of combination medications (Li X. et al., 2025).

This review aims to consolidate knowledge on the application of single-cell transcriptomics in the investigation of metastatic breast cancer. We examine the clinical problems and molecular heterogeneity associated with MBC, then critically assess the limits of bulk RNA-seq methodologies. We subsequently emphasize contemporary breakthroughs in single-cell technologies and their implications in MBC research, encompassing investigations into immunological heterogeneity, stromal remodeling, and metastatic progression. Finally, we delineate prospective avenues and unresolved inquiries, highlighting the potential of single-cell methodologies to reconcile tumor complexity with precision oncology.

## 2 Fundamentals of single-cell transcriptomics

The scRNA-seq encompasses a range of experimental platforms and analytical pipelines that allow the capture and transcriptional profiling of individual cells. The two principal strategies for cell capture are plate-based methods (e.g., Smart-seq2) and droplet-based methods (e.g., 10x Genomics Chromium, Drop-seq). Plate-based approaches provide full-length transcript coverage with high sensitivity but are typically lower throughput and more costly. In contrast, droplet-based systems enable high-throughput profiling of tens of thousands of cells in a single run, albeit with reduced coverage limited to the 3′or 5′ends of transcripts. This approach addresses the constraints of bulk RNA-seq, which obscures the diversity and functional adaptability of cells inside a tumor by averaging transcriptome data across different populations (Tzec‐Interián et al., 2025). Beyond these, microwell-based (e.g., Seq-Well) and combinatorial indexing (e.g., SPLiT-seq, sci-RNA-seq) approaches have expanded accessibility, offering cost-effective and scalable solutions, albeit with trade-offs in sensitivity and cell recovery.

For data analysis, scRNA-seq typically follows a pipeline that includes quality control, normalization, dimensionality reduction (e.g., PCA, UMAP, t-SNE), clustering, and differential expression analysis. Advanced tools, such as Monocle, Slingshot, and RNA velocity, enable trajectory inference and lineage reconstruction. Meanwhile, CellPhoneDB and NicheNet facilitate the study of intercellular communication. The procedure encompasses the isolation of individual cells, extraction of their mRNA, reverse transcription into cDNA, amplification of the cDNA, and execution of high-throughput sequencing (Camperi et al., 2025). Within the framework of MBC, scRNA-seq has been pivotal in identifying unusual subclones, therapy-resistant persister cells, and alterations in the immune milieu across primary and metastatic lesions. Each platform and method carries distinct advantages and limitations. For example, Smart-seq2 excels in isoform detection but lacks scalability; droplet-based methods are optimal for capturing cellular diversity but are sensitive to dropout events. Earlier studies provide comprehensive overviews of the technical differences, strengths, and challenges (Baker et al., 2025).

### 2.1 Key platforms and technologies

The scRNA-seq in MBC encompasses both droplet-based and full-length transcriptomic methodologies. The 10x Genomics Chromium platform is the predominant research choice due to its scalability, cost efficiency, and compatibility with fresh tumor samples and circulating tumor cells (CTCs) (Saw and Song, 2025). Clinical investigations by Huang et al. employed 10x Genomics to investigate immune exhaustion and niche-specific transcriptional states in metastatic liver and brain lesions (Huang et al., 2025). In preclinical studies, Zhang et al. utilized 10x Genomics on mouse xenograft models to delineate resistance to HER2-targeted treatments (Zhang W. et al., 2025). Alternatively, Smart-seq2 and Smart-seq3, which provide comprehensive transcript coverage, have been employed in investigations that necessitate accurate isoform identification and splicing analysis, as exemplified by Baker et al., who characterized uncommon subpopulations in primary and metastatic cancers (Baker et al., 2025). Innovative methods, such as Drop-seq, Seq-Well, and 10x Visium, have demonstrated applicability in clinical research, including spatially resolved transcriptomics, as utilized by Sun et al. to delineate the distribution of immune cells (Sun et al., 2025).

### 2.2 Data analysis pipelines and bioinformatics tools

The scRNA-seq research is computationally demanding and comprises several essential phases. The initial preprocessing is aligning reads to a reference genome (utilizing tools such as STAR or CellRanger), filtering out low-quality cells, and performing normalization (Bhattacharya, 2025). Dimensionality reduction techniques (e.g., PCA, t-SNE, UMAP) facilitate the display of intricate datasets, whereas clustering methods like Louvain or Leiden assist in identifying discrete cell populations. Functional annotation is often conducted using methods such as SingleR or CellTypist, whereas differential gene expression analysis identifies indicators indicative of resistance or immunological dysfunction (Almahdi et al., 2025). In metastatic scenarios, trajectory inference (utilizing Monocle, Slingshot, or PAGA) has been employed to describe the temporal progression of tumor clones and immune cell states (Ma H. et al., 2025). Inference of cell-cell communication with CellPhoneDB, NicheNet, or iTALK has elucidated ligand-receptor interactions between tumor cells and stromal or immune cells within metastatic habitats (Gao et al., 2022).

However, scRNA-seq encounters various technical and biological constraints, especially in MBC. A significant challenge is the low efficiency of RNA capture and elevated dropout rates, resulting in the underrepresentation of specific transcripts, particularly in rare or sensitive cells (Manzoor et al., 2025). Tumor dissociation procedures may create bias, frequently resulting in the under-sampling of immune or stromal compartments essential for metastatic colonization. Clinically, the availability of samples is constrained by the invasive nature of obtaining metastatic biopsies from organs such as the brain, liver, or bone (Hricak et al., 2025). Moreover, single-cell research generates high-dimensional data, necessitating sophisticated bioinformatics proficiency and computer resources. Investigations by Roostee identified constraints in patient cohort size and the lack of spatial or multi-omics integration, which may compromise biological resolution and clinical generalizability (Roostee, 2025).

## 3 Tumor heterogeneity in metastatic breast cancer

Tumor heterogeneity is a hallmark characteristic of MBC, significantly impacting disease development, treatment resistance, and clinical outcomes. At the center of this heterogeneity is intratumoral clonal variety, characterized by the coexistence of numerous genetically and phenotypically diverse cell populations within a single tumor. These subclones may differ in their proliferation rates, medication sensitivities, and metastatic capabilities, which complicates successful treatment (Zhang and Wang, 2025). Furthermore, EMT fosters dynamic heterogeneity by allowing epithelial tumor cells to adopt mesenchymal characteristics, increasing invasiveness and metastatic spread (Ghafoor et al., 2025). This phenotypic flexibility, influenced by transcriptional reprogramming and tumor microenvironmental signals, enables cancer cells to alternate between proliferative and migratory states, complicating therapeutic targeting (Kumar. 2024).

The interaction between primary and metastatic tumor locations further intensifies the diversity in MBC. Metastatic lesions frequently display genetic divergence from the initial tumor due to clonal evolution influenced by selective factors, including medication and immune surveillance (Zhang and Wang, 2025). Subclones exhibiting survival advantages in the metastatic niche may show markedly different receptor status, gene expression, and signaling pathways compared to their progenitor cells. This spatial heterogeneity presents difficulties for precision treatment, as a primary tumor biopsy may not adequately reflect the metastatic landscape (Lu et al., 2025). Moreover, organ-specific microenvironments, such as those found in the bone, liver, or brain, impose distinct selective pressures that influence the phenotypes of metastatic cells, underscoring the necessity for site-specific therapeutic strategies (Dawalibi et al., 2025).

**FIGURE 1:**
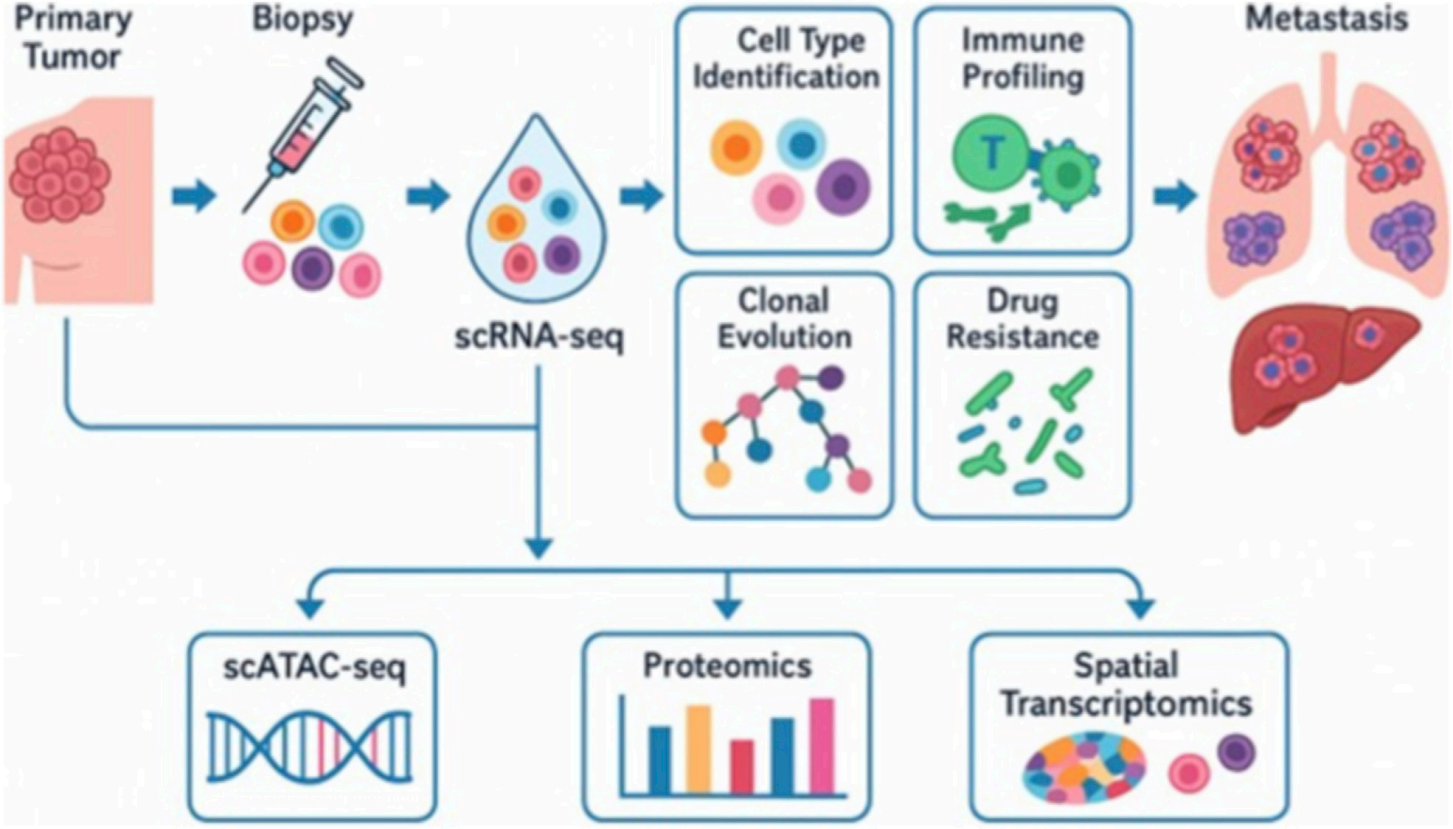
The diagram illustrates the workflow and major research applications of scRNA-seq. Following tumor biopsy, individual cells undergo single-cell gene expression profiling. scRNA-seq enables the identification of cell types, immune profiling, mapping of clonal evolution, and the detection of drug-resistant populations. These applications provide insights into tumor heterogeneity, therapy response, and the biological processes underlying metastasis. Integration with complementary platforms such as scATAC-seq (chromatin accessibility), proteomics, and spatial transcriptomics further enhances the resolution of tumor biology. Moreover, it facilitates the study of metastatic adaptations in distant organs. Notably, the figure emphasizes that scRNA-seq serves as a tool for studying metastasis rather than driving it.

Furthermore, CTCs also contribute to this evolutionary process by disseminating from the primary tumor into the bloodstream. Although their functional roles and transcriptional heterogeneity are better elaborated in later sections, it is important to note here that CTCs represent a key reservoir of tumor diversity that sustains metastatic seeding. Recent advancements in single-cell transcriptomics have elucidated the degree of molecular subtype plasticity in MBC, revealing how tumor cells can transition among established breast cancer subtypes, including Luminal A/B, HER2-enriched (Ferrer et al., 2025), and triple-negative (TNBC) states (Jie et al., 2025). For example, scRNA-seq studies have demonstrated that within tumors categorized as a single subtype using bulk profiling, cells may exhibit mixed or hybrid expression patterns, indicating dynamic interconversion between phenotypes (Yan et al., 2024). This flexibility can facilitate resistance to targeted therapies, such as HER2+ cells evolving into a TNBC-like phenotype, or enable cells to avoid immune recognition (He Y. et al., 2025). These findings underscore the importance of single-cell resolution in understanding how heterogeneity drives metastasis and in informing more personalized and adaptable treatment approaches. Figure 1 illustrates the diverse applications of scRNA-seq MBC, including the characterization of tumor heterogeneity, identification of metastatic cell populations, and mapping of the tumor immune microenvironment.

## 4 Mapping tumor evolution through single-cell profiling

The scRNA-seq facilitates gene expression profiling at an individual cell level, allowing researchers to deduce lineage links among tumor cells and construct phylogenetic trees that illustrate clonal diversification over time (Li S. et al., 2025). These reconstructions elucidate the emergence, coexistence, and evolution of genetically different subclones under the selective constraints of treatment and immune surveillance. This technique in MBC has revealed early branching events from the primary tumor that generate metastatic subclones with distinct transcriptional profiles (Joint, 2024). The integration of single-cell DNA sequencing (scDNA-seq) and copy number variation (CNV) analysis significantly improves the resolution of lineage maps, enabling researchers to associate genetic alterations with functional phenotypes, including drug resistance, immune evasion, and EMT (Zhang and Wang, 2025).

**FIGURE 2:**
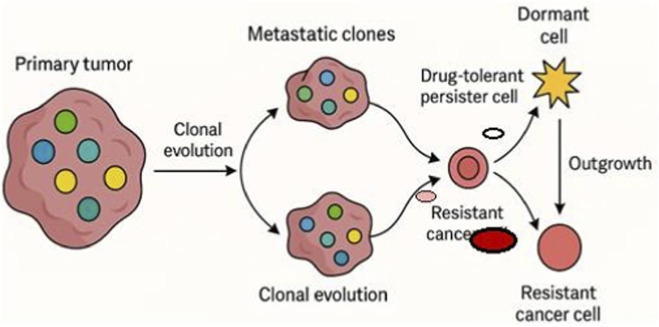
Tumor evolution and resistance. This schematic illustrates clonal dynamics in metastatic breast cancer leading to progression and therapy resistance. A primary tumor with genetically and phenotypically distinct subclones (colored circles) undergoes clonal evolution. During metastatic spread, selective subclones predominate, resulting in metastatic lesions with altered compositions compared to the primary tumor. Within these metastatic clones, some cells may undergo transcriptional reprogramming or metabolic adaptation to become drug-tolerant persister cells (smaller pale-red circles), which can either transition into dormant cells (star-shaped) or evolve into resistant cancer cells (bold red circles). Resistant clones can outgrow and repopulate the tumor mass, driving relapse and disease progression. Each cell state is schematically represented with distinct symbols for clarity. Single-cell transcriptomic approaches can map these dynamic transitions, highlighting cellular plasticity, therapy-induced selection, and microenvironmental adaptation in the evolution of resistance.

In addition to clonal lineage tracing, scRNA-seq offers significant insights into the temporal and spatial dynamics of metastasis. Tumor cells can disperse early and remain dormant or evolve concurrently at distant locations, resulting in metastatic lesions with characteristics distinct from the parent tumor (Bhattacharya et al., 2025). Single-cell profiling of corresponding primary and metastatic samples has revealed site-specific transcriptional modifications; for instance, brain metastases frequently exhibit neurotropic and immunosuppressive signatures that are absent in the primary tumor (Lu et al., 2025). Moreover, scRNA-seq of CTCs has demonstrated that these cells often inhabit transitional states, balancing proliferative and migratory roles, and may act as real-time indicators of changing tumor clones (Santamaria et al., 2025). These findings collectively emphasize the intricate and dynamic characteristics of metastatic spread, underscoring the need for longitudinal, multi-site single-cell research to fully capture tumor growth. The clonal dynamics of metastatic breast cancer are illustrated in Figure 2, emphasizing the role that changing subclonal populations play in tumor growth and the emergence of treatment resistance.

In a pivotal study by van Roey et al., utilizing patient-derived xenograft (PDX) models, researchers demonstrated that transcriptional plasticity associated with EMT facilitated therapeutic evasion and metastatic spread. Moreover, HER2-targeted therapeutic resistance in mouse xenografts was examined, revealing divergent evolutionary pathways and the formation of drug-tolerant subpopulations (Van Roey et al., 2021). Muhmed et al. expanded upon these findings by reviewing spontaneous metastasis models in mice, uncovering early transcriptional divergence between primary and metastatic lesions, with metabolic reprogramming identified as a hallmark of metastatic start (Mohamed, 2025). These models, albeit non-human, closely replicate the spatial and temporal dynamics of tumor evolution, facilitating the analysis of lineage-specific resistance and site-specific adaptations in preclinical environments. On the other hand, clinical scRNA-seq investigations have demonstrated tumor development and therapeutic response in human MBC patients with significant translational value. Laisné et al. performed longitudinal single-cell profiling of breast cancer patients receiving chemotherapy and discovered the formation of quiescent DTP cells that remained after treatment (Laisné et al., 2025). Han et al. investigated niche-specific adaptation in liver and brain metastases, revealing transcriptional pathways distinct to each metastatic location (Han et al., 2025). Furthermore, Sudupe et al. combined spatial transcriptomics with single-cell RNA sequencing in multi-region biopsies to delineate immune fatigue gradients and spatial clonal heterogeneity (Sudupe et al., 2024). Table 1 summarizes preclinical and clinical scRNA-seq studies in metastatic breast cancer, including study models, sample sources, platforms, and key findings that have improved our understanding of tumor heterogeneity, immune dynamics, and therapeutic resistance.

**TABLE 1:** Overview of principal preclinical and clinical scRNA-seq investigations in metastatic breast cancer.

Study type	Subtype/Metastatic site	Sample size	Platform	Main objective	Key findings	Limitations	Ref
Preclinical (PDX)	Lung metastases (PDX)	6 mice	10x Genomics	EMT resistance	EMT-related clones	No human data	Saw and Song (2025)
Preclinical	Liver metastases	5 mice	10x Genomics	Resistance mapping	Escape routes for HER2 treatment	Absence of immune context	Zhang et al. (2025b)
Preclinical	Metastatic breast organoids	Organoid cultures	Smart-seq3	Cell plasticity	Metabolic alterations and reprogrammed clones	No confirmation from the patient	Huang et al. (2025)
Preclinical	Spontaneous metastases (mouse)	Rodent model	10x Genomics	Metastatic seeding	Initial divergence and metabolic change	No human samples	Zhang and Wang (2025)
Clinical	Serial samples, or CTCs	16 Patients	10x Genomics	CTC evolution	Markers of dynamic resistance	No paired tumor biopsies	Yadav et al. (2025)
Clinical	Bone metastases (HR+)	15 patients	10x Genomics	Immune-stromal interactions	Inhibition of the immune system and fibroblast activity	No response tracking	Griffiths et al. (2025)
Clinical	TNBC pleural metastases	9 patients	10x Genomics	TNBC heterogeneity	Plasticity and uncommon subpopulations	Absence of spatial validation	Yan et al. (2024)
Clinical	Metastatic lesions in many regions	20 patients	10x + Spatial	The immune milieu	Immune fatigue in space	High cost	Zhang and Wang (2025)
Clinical	Liver and brain metastasis	18 patients	10x Genomics	Niche-specific changes	Tissue-specific gene expression	Limited samples	Huang et al. (2025)
Clinical	Diverse (pre/post-chemotherapy)	15 patients	10x Genomics	Chemotherapy response	Persistent cell states	Moderate size	Griffiths et al. (2025)
Clinical	CTCs + Biopsies	19 patients	Drop-seq	Immune landscape	Immune suppression and T cell depletion	No therapy link	Sun et al. (2025)
Clinical	Mixed (Primary + Metastatic)	11 patients	Smart-seq2	Tumor heterogeneity	immunological escape and tumor cells that resemble stems	Small sample, no follow-up	Tang et al. (2025)

MBC (*Metastatic Breast Cancer),* scRNA-seq (*Single-Cell RNA, Sequencing)*, PDX (*Patient-Derived Xenograft),* CTCs (*Circulating Tumor Cells),* EMT (*Epithelial–Mesenchymal Transition)*, TNBC (*Triple-Negative Breast Cancer),* HR+ (*Hormone Receptor-Positive), 10x Genomics* (droplet-based high-throughput platform), *Drop-seq* (Microfluidics-based platform), *Smart-seq2/3* (full-length RNA-sequencing techniques).

## 5 The TME and immune landscape

The tumor microenvironment in MBC is a multifaceted ecosystem consisting of malignant epithelial cells, stromal fibroblasts, endothelial cells, and a heterogeneous array of immune cells. In contrast to original tumors, metastatic lesions frequently display unique modifications in tumor microenvironment composition, influenced by organ-specific niches such as bone, liver, lung, or brain (Oh et al., 2025). Abundant tissue-resident macrophages and immunosuppressive fibroblasts characterize liver and brain metastases, whereas bone metastases often entail osteoclast-mediated remodeling and immune exclusion (Griffiths et al., 2025). Studies utilizing scRNA-seq, including Li et al., have demonstrated that the metastatic TME is characterized by diminished infiltration of cytotoxic T cells and an elevated presence of cancer-associated fibroblasts (CAFs), myeloid-derived suppressor cells (MDSCs), and regulatory T cells (Tregs), all of which facilitate immune evasion and tumor advancement (Mei et al., 2024).

The scRNA-seq has been essential in elucidating the intricate states of immune cells within the metastatic milieu. Casalegno et al. have demonstrated that cytotoxic CD8^+^ T cells in metastatic lesions often exhibit transcriptional signatures indicative of exhaustion, characterized by the upregulation of immune checkpoint markers, including PD-1, LAG-3, and TIGIT (Casalegno Garduño et al., 2025). Simultaneously, tumor-infiltrating natural killer (NK) and dendritic cells exhibit diminished activation and cytokine signaling patterns (Li F. et al., 2025). Additionally, tumor-associated macrophages (TAMs) in metastatic locations have M2-like behaviors that facilitate tumor survival and angiogenesis. These single-cell analyses offer a detailed perspective on the immunological dysfunctions present in MBC, which are frequently obscured in bulk RNA-seq research (Aleebrahim-Dehkordi et al., 2023). The interactions between tumor cells and stromal/immune elements are pivotal in influencing the progression and therapeutic resistance of MBC. Ligand-receptor analysis utilizing tools such as CellPhoneDB and NicheNet has revealed critical signaling pathways that facilitate tumor-stroma interactions in scRNA-seq investigations (Sarkar et al., 2025). Interactions between CAFs and tumor cells, mediated through TGF-β and IL-6 signaling pathways, reduce immune suppression and promote EMT (Li Y. et al., 2025). Similarly, reciprocal interactions between tumor cells and fatigued T cells, mediated by PD-L1/PD-1 signaling, further inhibit anti-tumor immunity. Spatially resolved scRNA-seq tools, such as 10x Visium, have enhanced these discoveries by maintaining tissue architecture and uncovering discrete zones of immune suppression inside the metastatic niche (Dai et al., 2024). These results collectively change our comprehension of the tumor microenvironment and present novel opportunities for immune-targeted treatments in metastatic breast cancer.

### 5.1 Unique insights from single-cell approaches

Unlike bulk or IHC assays, single-cell transcriptomics enables the delineation of exhaustion trajectories in CD8^+^ T cells, identifying intermediate states that predict responsiveness to immune checkpoint blockade (Zemanek et al., 2023). Single-cell approaches have uncovered hybrid Tumor-Associated Macrophages (TAMs) states that simultaneously express pro-inflammatory (IL-1β, TNF) and suppressive (IL-10, TGF-β1) programs, illustrating a functional plasticity not resolved by traditional M1/M2 classification (Alexander et al., 2021). Single-cell transcriptomics distinguished multiple Cancer-Associated Fibroblasts (CAF) subpopulations, including antigen-presenting CAFs that activate PD-L2–mediated T cell suppression, which would remain hidden in bulk analyses (Milosevic and Östman, 2024). Spatially resolved single-cell analyses revealed that PD-L1^+^ tumor cells cluster in perivascular niches enriched with exhausted T cells. This spatial relationship cannot be captured by flow cytometry or bulk RNA sequencing (Yang et al., 2023). Ligand–receptor inference from single-cell data revealed CAF–T cell and TAM–T cell signaling axes, pinpointing immunosuppressive communication networks invisible to traditional profiling (Qin et al., 2023). Single-cell transcriptomics has identified rare endothelial subsets that express both angiogenic factors and checkpoint ligands, highlighting their dual role in vascular remodeling and immune evasion (Wen, 2024).

## 6 Mechanisms of therapeutic resistance uncovered by scRNA-seq

**FIGURE 3:**
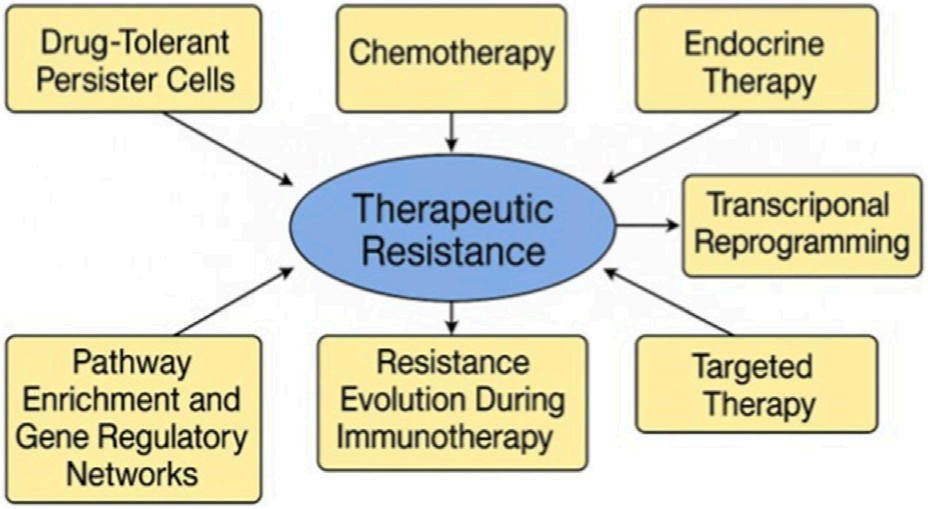
Mechanisms of Therapeutic Resistance in Metastatic Breast Carcinoma Revealed by single-cell RNA sequencing. This figure illustrates the primary molecular and cellular pathways that contribute to therapy resistance in metastatic breast cancer, as determined through scRNA-seq. The map highlights the evolution of resistance via drug-tolerant persister cells, transcriptional reprogramming, immunological evasion during immunotherapy, and adaptive modifications to chemotherapy, endocrine, and targeted therapies. Pathway enrichment and gene regulatory network reconfiguration highlight the continuous emergence of resistance within the tumor microenvironment.

Preclinical studies have yielded essential insights into the existence and behavior of drug-tolerant persister (DTP) cells by scRNA-seq in breast cancer models. In a study by Griffiths et al., a subpopulation of DTP cells was identified in TNBC mice that persisted after chemotherapy, exhibiting mesenchymal characteristics and activation of lipid metabolism pathways (Griffiths et al., 2025). A further study by Chalabi et al. on melanoma, although not specifically related to breast cancer, provided a transportable framework by illustrating the emergence of DTPs through non-genetic transcriptional reprogramming (Chalabi, 2024). Similarly, Liu et al. employed single-cell methodologies in HER2+ breast cancer xenografts to monitor the development of treatment-resistant clones, demonstrating that these cells entered a quiescent, slow-cycling state capable of subsequently inducing relapse (Liu et al., 2025). These results collectively affirm the efficacy of scRNA-seq in identifying unusual, pre-resistant traits that elude traditional detection approaches and indicate potential treatment failure. Figure 3 illustrates the critical molecular and cellular mechanisms underlying epithelial-mesenchymal transition, immune evasion, and the activation of survival signaling cascades in metastatic breast cancer therapeutic resistance.

Mouse models and PDXs have demonstrated transcriptional adaptation as a primary mechanism of resistance to chemotherapy and hormone therapy. Dawoud et al. utilized scRNA-seq on PDXs subjected to paclitaxel treatment and noted transcriptional alterations indicative of EMT-like and inflammatory states in the surviving cells (Liu et al., 2025). In ER + breast cancer, Crowley et al. indicated that endocrine-resistant cells diminished estrogen receptor signaling. Still, they compensated via activation of the PI3K/AKT pathway, a conclusion corroborated by scRNA-seq analysis of resistant clones (Dawoud et al., 2023). Clinical biopsies from neoadjuvant trials corroborated these findings, as scRNA-seq demonstrated that surviving tumor cells exhibited hybrid epithelial–mesenchymal states and immune-evasive behaviors after therapy. These modifications enhanced resistance and reconfigured the tumor microenvironment, enabling resistant clones to circumvent therapeutic and immunological constraints (Crowley, 2022).

Single-cell transcriptomics has been used to investigate the progression of resistance to immunotherapy in clinical environments. Coleman et al. conducted a crucial study examining tumor-infiltrating lymphocytes (TILs) in patients with TNBC. They discovered that individuals exhibiting significant clonal diversity and cytotoxic characteristics demonstrated greater responsiveness to immune checkpoint inhibitors (Feng et al., 2025). In comparison, Nair et al. employed scRNA-seq on breast cancer metastases to establish that T cell exhaustion intensified after treatment, characterized by the overexpression of PD-1, LAG-3, and TIM-3 in TILs (Coleman et al., 2025). In preclinical models, He et al. delineated the progression of fatigued CD8^+^ T cells after anti-PD-1 therapy and demonstrated how resistant cancers reconfigured their immune microenvironments via macrophage recruitment and IFN signaling (Nair et al., 2025). These findings underscore the dynamic characteristics of immune evasion and the necessity for longitudinal monitoring of immune cell states throughout treatment.

Regarding regulatory networks and pathway enrichment, preclinical and clinical investigations have utilized computational tools such as SCENIC, Monocle, and CellPhoneDB to deduce resistance-associated transcription factors and signaling connections (He L. et al., 2025). Fatima et al. examined breast cancer metastases and identified ZEB1 and SOX9 as primary regulators of EMT and resistance to HER2 suppression (Wang N. et al., 2024). Likewise, Zeng et al. developed pseudotime trajectories in head and neck malignancies to simulate transcriptional changes during therapy resistance, which has subsequently influenced analogous methodologies in breast cancer (Famta et al., 2025). In ER + PDX models, Yu et al. identified an enrichment of the Notch and IL-6/STAT3 pathways in endocrine-resistant cells. These investigations demonstrate that scRNA-seq identifies resistant cellular states and delineates actionable biochemical pathways, facilitating the development of sensitive medication combinations for both the cell and its adaptive signaling environment (Zeng, 2024). Table 2 lists scRNA-seq-identified therapeutic resistance mechanisms in metastatic breast cancer, including major pathways, cell types, therapeutic contexts, and supporting studies that demonstrate how cancers evade single-cell treatment.

**TABLE 2:** scRNA-seq-identified mechanisms of therapeutic resistance in metastatic breast cancer.

Resistance mechanism	Cell types involved	Sample source/Model	Therapy type	Pathways/Genes involved	Cellular/Molecular features	Clinical implication	Ref
Niche-specific adaptation	Metastatic subclones	Liver, brain, and bone mets	Diverse therapeutic modalities	TCF4, APOE, and CXCR4	Programs for site-specific expression	Response to Differential Therapy by Organ	Han et al. (2025)
DNA repair reprogramming	HR + tumor, cells with TP53 mutations	Organoids generated from patients	Chemotherapy, PARP inhibitors	CHK1, RAD51, and BRCA1	Reactivation of the BRCA1/2 pathway	Resistance to treatments that damage DNA	Yu et al. (2025)
Microenvironmental modulation	MDSCs, CAFs	MBC biopsies	Diverse therapeutic modalities	CXCL12, TGFB1, and IL6	IL-6 signaling, TGF-β, and CAF activation	Immunoevasion and stromal protection	Li et al. (2025d)
Metabolic adaptation	Tumor cells that resemble stem cells	Liver and bone metastases	Targeted therapies	SCD1, CPT1A, and PGC1A	Increased metabolism of fatty acids and OXPHOS	SCD1, CPT1A, and PGC1A	Zhang et al. (2025c)
EMT-associated resistance	tumor cells that resemble basal cells	Brain metastases and PDX	Anti-HER2, Endocrine	TWIST1, SNAI1, and ZEB1	E-cadherin loss and ZEB1 overexpression	Resistance to anti-HER2 treatment and metastasis	Wang et al. (2024a)
Immune cell exhaustion	NK cells and CD8^+^ T cells	Biopsies and CTCs	Immunotherapy	TIGIT, LAG3, and PDCD1	TIGIT, LAG-3, and PD-1 overexpression	Immune checkpoint blockade failure	Nair et al. (2025)
Transcriptional reprogramming	Stromal cells and tumors	Biopsies and organoids	Chemotherapy that targets HER2	TWIST1, HIF1A, and MYC	Chromatin remodeling, MYC signaling, and EMT	Resistance to therapy and adaptive switching	Liu et al. (2025)
Drug-tolerant persister cells	Tumor cells in the epithelium	Tumors in MBC patients	Endocrine, Chemotherapy	BCL2, SOX9, and NR2F1	Signaling for survival and quiescence	Relapse risk and treatment persistence	Chalabi (2024)

MBC (Metastatic Breast Cancer), scRNA-seq (Single-Cell RNA, Sequencing), PDX (Patient-Derived Xenograft), CTC (Circulating Tumor Cell), CAF (Cancer-Associated Fibroblast), MDSC (Myeloid-Derived Suppressor Cell), HR+ (Hormone Receptor-Positive), EMT (Epithelial–Mesenchymal Transition), OXPHOS (Oxidative Phosphorylation), PD-1 (Programmed Cell Death Protein 1), TIGIT (T-cell Immunoreceptor with Ig and ITIM, Domains), LAG-3 (Lymphocyte-Activation Gene 3). TWIST1 (Twist Family BHLH, Transcription Factor 1), SNAI1 (Snail Family Transcriptional Repressor 1), ZEB1 (Zinc Finger E-box-Binding Homeobox 1), MYC (Myelocytomatosis Viral Oncogene Homolog), HIF1A (Hypoxia-Inducible Factor 1-alpha), SCD1 (Stearoyl-CoA, Desaturase-1), CPT1A (Carnitine Palmitoyltransferase 1A), PGC1A (Peroxisome Proliferator-Activated Receptor Gamma Coactivator 1-alpha). CXCL12 (C-X-C Motif Chemokine Ligand 12), TGFB1 (Transforming Growth Factor Beta 1), IL6 (Interleukin 6), RAD51 (RAD51 Recombinase), CHK1 (Checkpoint Kinase 1), BRCA1/2 (Breast Cancer Genes 1 and 2), CXCR4 (C-X-C Chemokine Receptor Type 4), TCF4 (Transcription Factor 4), APOE (Apolipoprotein E).

## 7 Liquid biopsy and CTCs in MBC

In preclinical models, liquid biopsy has evolved as a minimally invasive technique to capture dynamic alterations in tumor progression, particularly by extracting and studying CTCs. Mouse models of breast cancer have shown that CTCs are not only passive indicators of disease load but active participants in metastasis (Stouras et al., 2023). Yadav et al. indicate that circulating tumor cells frequently display hybrid epithelial–mesenchymal characteristics, which facilitate their survival in the bloodstream and enable them to colonize distant organs. Researchers have utilized scRNA-seq to elucidate the transcriptional fingerprints of CTCs associated with stemness, drug tolerance, and immune evasion (Mishra et al., 2025). Preclinical research by Lan et al. demonstrated that CTCs undergo dynamic transitions between epithelial and mesenchymal forms, a mechanism that imparts resistance to chemotherapy and immune-mediated elimination (Yadav et al., 2025). These data corroborate the concept that CTCs serve as a reservoir of phenotypic flexibility, enabling them to facilitate metastasis and recurrence.

Clinically, liquid biopsy is a valuable real-time instrument for assessing disease progression and treatment response in patients with MBC. Multiple studies have demonstrated that the presence, abundance, and molecular features of CTCs are associated with poor prognosis and therapy resistance (Lan et al., 2025). In the STIC CTC trial, CTC enumeration informed treatment decisions in hormone receptor-positive metastatic breast cancer, yielding outcomes analogous to conventional clinical evaluations (Rapanotti et al., 2024). More advanced investigations have employed single-cell transcriptome profiling of patient-derived CTCs, revealing several subpopulations enriched for EMT markers, immune checkpoint ligands (such as PD-L1), and stemness-associated genes (Ma S. et al., 2025). Zaky et al. further demonstrated that CTC transcriptomes reflect intra-tumoral heterogeneity and predict resistance to HER2-targeted therapies (Capuozzo et al., 2023). Collectively, these findings establish scRNA-seq of CTCs as a clinical interface of tumor biology, enabling non-invasive monitoring of tumor evolution and guiding personalized therapy in MBC.

## 8 Integration of single-cell transcriptomics with other omics

Preclinical models have demonstrated how the integration of multi-omics at single-cell resolution can enhance our understanding of metastatic breast cancer. In mouse models and PDXs, integrating scRNA-seq with scATAC-seq has revealed the epigenetic processes that govern cellular plasticity and treatment resistance (Zaky et al., 2025). For instance, in a PDX model of endocrine-resistant ER + breast cancer, researchers combined scRNA-seq with chromatin accessibility data, demonstrating that resistant tumor cells display heightened enhancer activity near transcription factors such as FOXC1 and SOX9, which promotes a stem-like phenotype (Dawoud et al., 2023). Similarly, preclinical applications of CITE-seq have analyzed cell surface protein expression together with gene transcription, revealing phenotypically distinct immune and stromal populations that facilitate tumor growth (Olive et al., 2025). Preclinical multi-omics initiatives have identified metabolic vulnerabilities by correlating scRNA-seq with single-cell metabolomics, revealing that DTP cells exhibit increased oxidative stress tolerance and enhanced lipid metabolism. This observation has prompted trials of metabolic inhibitors (Jacek, 2025).

In comparison, clinical integration of single-cell transcriptomics with epigenetic and proteomic data from patient biopsies has elucidated critical insights into the heterogeneity of metastatic breast cancer (Guan and Quek, 2025). For example, in fresh biopsies from MBC patients, single-nucleus ATAC-seq (snATAC-seq) combined with scRNA-seq revealed correlations between open chromatin regions and the expression of drug-resistance genes, including those associated with PI3K/AKT signaling (El Gazzah et al., 2025). In HER2+ and triple-negative subtypes, integrated investigations have revealed transcriptional pathways enriched in interferon response and glycolysis, concomitant with chromatin remodeling and the loss of epithelial markers, underscoring prospective targets for combination treatments (Wang N. et al., 2024).

Furthermore, investigations integrating mass cytometry (CyTOF) and scRNA-seq in corresponding tumor and blood specimens have identified functionally distinct T cell states, associated with fatigue, activation, or cytotoxicity profiles that would remain indistinguishable using transcriptomic data alone (Caravan, 2025). The application of spatially resolved transcriptomics in both preclinical murine models and clinical patient samples has significantly enhanced the interpretation of single-cell discoveries within their anatomical context. In mouse models, spatial transcriptomics has been employed to delineate immune cell exclusion zones within metastatic lung lesions, revealing that tumor cells enhance the expression of checkpoint ligands, such as PD-L1, near exhausted T cells (Shang et al., 2025). In patient-derived brain metastasis samples, analysis of breast cancer brain metastasis samples using 10x Visium and NanoString GeoMx demonstrated geographic variability in immune suppression, characterized by perivascular niches populated by macrophages and tumor cells that express neuroinflammatory genes, including CXCL10 and IL-6 (Kang et al., 2025). These methodologies maintain the spatial arrangement of tissues while elucidating transcriptional complexity, providing essential insights into the influence of local microenvironments on tumor behavior (Kzhyshkowska et al.). Ultimately, these multi-omics approaches hold promise for elucidating the determinants of site-specific metastasis and resistance, thereby facilitating the development of genuinely tailored therapeutic strategies.

## 9 Clinical translation and therapeutic implications

As our understanding of tumor heterogeneity advances, scRNA-seq is poised to revolutionize patient stratification models in oncology. In metastatic breast cancer, when histologically analogous tumors demonstrate varied clinical behavior, single-cell profiling can differentiate patients based on the existence of therapy-resistant clones, immune-suppressive microenvironments, or aggressive mesenchymal-like cellular states (Okoh). By identifying these therapeutically pertinent subpopulations, scRNA-seq facilitates the selection of customized therapies, such as the combination of immune checkpoint inhibitors with medicines targeting stromal or myeloid compartments in patients with immunosuppressive tumor microenvironments (Tomanelli, 2025). Moreover, scRNA-seq can help identify individuals who are likely to benefit from reduced treatment regimens, thereby minimizing toxicity while maintaining efficacy in low-risk populations (Li W. et al., 2025).

Identifying predictive biomarkers with scRNA-seq has accelerated, facilitating real-time and retrospective evaluations of therapy efficacy. Clinical single-cell studies, unlike preclinical bulk approaches, can identify rare yet functionally significant cell types that contribute to recurrence or resistance (Ling et al., 2025). Signatures linked to T cell exhaustion, M2-like macrophage enrichment, or epithelial-mesenchymal transition transcriptional programs have been suggested to indicate inadequate responses to immunotherapy or chemotherapy (Sabit et al., 2025b). Moreover, clinical single-cell analysis of CTCs offers a non-invasive method for monitoring dynamic biomarker expression during therapy, allowing clinicians to detect the formation of resistant phenotypes before they manifest radiologically or clinically. These prediction capabilities may soon guide adaptive therapy algorithms in metastatic contexts (Xu et al., 2025).

Numerous translational and clinical research initiatives incorporate scRNA-seq to connect laboratory findings with therapeutic applications. Clinical trials, such as the I-SPY2 trial and the Human Tumor Atlas Network (HTAN), produce extensive single-cell datasets to identify response predictors and mechanisms of resistance in actual patient populations (Wang X. et al., 2024). Furthermore, cancer hospitals in Europe and the United States have initiated pilot programs employing scRNA-seq to inform therapy selection in challenging or relapsed metastatic cases, especially in triple-negative or HER2-low subtypes (Paul et al., 2025). With decreasing sequencing costs and more efficient data pipelines, integrating single-cell profiling into clinical workflows, potentially in conjunction with genomic and proteomic assays, may provide a comprehensive understanding of tumor biology, facilitating genuinely personalized, evolution-informed treatment approaches for MBC (Turpin, 2024).

## 10 Future directions and challenges

This work identifies the restricted accessibility and consistency of high-quality metastatic tissue samples as a significant obstacle, which hinders the scalability of single-cell studies across various patient populations and metastatic locales (Vitorino, 2024). Numerous metastatic tumors pose challenges for biopsy due to anatomical or clinical limitations, and when samples are acquired, they often demonstrate inconsistent cellular viability, which undermines data integrity. The dynamic and varied characteristics of metastatic breast cancer hamper the long-term monitoring of clonal evolution and treatment response (Colonna, 2025). Although scientifically crucial, incorporating spatial context, epigenetic states, and multi-omic layers introduces considerable complexity to experimental design and subsequent analysis (Elshimy et al., 2025). Computational constraints persist, particularly in handling extensive datasets, reconciling batch effects, and elucidating transcriptional plasticity within evolving treatment environments (Yetgin, 2025).

Future goals for this field involve systematically integrating scRNA-seq and multi-omics into longitudinal clinical investigations in patients, while maintaining mechanistic validation in preclinical models, facilitated by enhanced minimally invasive techniques such as single-cell analysis of CTCs and liquid biopsies (Kwon and Joung, 2025). An increased focus on spatially resolved transcriptomics and its integration with artificial intelligence (AI) will be crucial for contextualizing single-cell data within the tumor microenvironment and predicting clinically relevant evolutionary trajectories (Coppola et al., 2025). Furthermore, establishing standardized pipelines, interoperable data repositories, and ethical frameworks for data sharing will be essential to facilitate collaborative, cross-institutional initiatives (Adekola, 2025). Ultimately, closing the gap between single-cell discoveries and practical clinical applications will require ongoing translational initiatives, multicenter validation studies, and adaptive clinical trial designs that consider tumor evolution as a critical factor in treatment (Adepoju and Adepoju, 2025).

## 11 Conclusion

In conclusion, single-cell transcriptomics has significantly enhanced our comprehension of the intricate cellular ecosystems that characterize metastatic breast cancer. By addressing tumor heterogeneity with unparalleled resolution, scRNA-seq has revealed the dynamic interactions among tumor cells, immunological populations, and stromal elements that contribute to metastasis and therapeutic resistance. Preclinical models have played a crucial role in identifying drug-tolerant persister cells, transcriptional plasticity, and immune evasion mechanisms, which have significantly transformed our understanding of tumor growth and treatment response. Complementary analyses of patient biopsies have confirmed the clinical relevance of these findings, highlighting resistant cellular subpopulations and immune-suppressive microenvironments in real-world metastatic contexts.

Moreover, preclinical integrations of scRNA-seq with additional omics modalities such as epigenomics, proteomics, and spatial transcriptomics have unveiled new opportunities for identifying predictive biomarkers and actionable molecular targets. Similarly, clinical studies using multi-omics profiling of patient-derived samples have begun to validate these targets and propose candidate biomarkers for therapy selection. Despite considerable obstacles in integrating these findings into standard clinical practice, the direction is evident: single-cell technologies are emerging as essential instruments in precision oncology. As the discipline advances, initiatives should enhance access to high-quality metastatic specimens, standardize data workflows, and create clinically validated, AI-integrated systems for patient classification and therapeutic decision-making. The incorporation of single-cell transcriptomics into longitudinal, patient-focused clinical studies, supported by mechanistic insights from preclinical models, has the potential to revolutionize the diagnosis, monitoring, and treatment of metastatic breast cancer, ushering in a new era of genuinely personalized and evolution-informed cancer therapy.
